# Device-Measured and Self-Reported Active Travel Associations with Cardiovascular Disease Risk Factors in an Ethnically Diverse Sample of Adults

**DOI:** 10.3390/ijerph18083909

**Published:** 2021-04-08

**Authors:** Katie Crist, Tarik Benmarhnia, Steven Zamora, Jiue-An Yang, Dorothy D. Sears, Loki Natarajan, Lindsay Dillon, James F. Sallis, Marta M. Jankowska

**Affiliations:** 1 Department of Family Medicine, UC San Diego, La Jolla, CA 92093, USA; dsears@health.ucsd.edu; 2 Herbert Wertheim School of Public Health and Human Longevity Science, UC San Diego, La Jolla, CA 92093, USA; tbenmarhnia@health.ucsd.edu (T.B.); lnatarajan@health.ucsd.edu (L.N.); ldillon@eng.ucsd.edu (L.D.); jsallis@health.ucsd.edu (J.F.S.); 3 Scripps Institution of Oceanography, UC San Diego, La Jolla, CA 92093, USA; 4 Qualcomm Institute/Calit2, UC San Diego, La Jolla, CA 92093, USA; s1zamora@eng.ucsd.edu (S.Z.); jayyang@eng.ucsd.edu (J.-A.Y.); 5 Department of Medicine, UC San Diego, La Jolla, CA 92093, USA; 6 College of Health Solutions, Arizona State University, 550 N 3rd Street, Phoenix, AZ 85004, USA; 7 Mary MacKillop Institute for Health Research, Australian Catholic University, Fitzroy, VIC 3065, Australia; 8 Population Sciences, Beckman Research Institute, City of Hope, 1500 E Duarte Rd, Duarte, CA 91010, USA; majankowska@ucsd.edu

**Keywords:** transportation, walking, biking, accelerometer, GPS, obesity, blood pressure, lipids, glucose, physical activity

## Abstract

Active travel (AT) provides an opportunity to alleviate the physical inactivity and climate crises contributing to the global chronic disease burden, including cardiovascular diseases (CVD). Though AT shows promising links to reduced CVD risk, prior studies relied on self-reported AT assessment. In the present study, device-measured and self-reported AT were compared across population subgroups and relationships with CVD risk biomarkers were evaluated for both measures. The study recruited an ethnically diverse sample (N = 602, mean age 59 years, 42% Hispanic/Latino ethnicity) from neighborhoods that varied by walkability and food access. AT was assessed using concurrently collected accelerometer and GPS data and self-report data from a validated survey. Relationships with body mass index (BMI), triglycerides, high-density lipoprotein (HDL) cholesterol, blood pressure (BP), and moderate-to-vigorous physical activity (MVPA) were modeled using multivariable linear regression. Devices captured more AT than did self-report. We found differences in AT measures by population subgroups, including race, ethnicity, education, income, vehicle access, and walkability. Men had more accelerometer-measured MVPA, though women self-reported more daily minutes. Both device and survey AT measures were positively associated with total accelerometer-measured MVPA, though the relationship was stronger with device-measured AT. Device-measured AT was associated with lower BMI. No other CVD risk biomarker was associated with either AT measure. No effect modification by Hispanic/Latino ethnicity was detected. Further studies with device-based measures are warranted to better understand the relationship between AT and cardiovascular health.

## 1. Introduction

Physical inactivity persists as a leading risk factor for premature mortality and chronic disease burden worldwide [1,2,3,4]. Cardiovascular diseases (CVD), though largely preventable, remain the leading cause of death globally [5]. Physical activity (PA) is effective in reducing CVD mortality as well as established CVD risk factors, including hypertension, dyslipidemia, poor glycemic control, and unhealthy body weight [2,6]. To achieve health benefits, it is recommended that adults accumulate at least 150 min per week of moderate-to-vigorous physical activity (MVPA) [2]. However, many adults fail to meet these guidelines [7]. Globally, it is estimated that roughly one quarter of adults do not meet MVPA recommendations using self-reported questionnaires, and in San Diego County, 14% of adults report no leisure-time PA at all [7,8,9].

Global warming presents a concurrent threat to global health. Increases in anthropogenic greenhouse gas (GHG) emissions have driven the global warming trend observed over the last century [10,11]. The transportation sector is one of the main contributors to GHG emissions globally [12]. In California, transportation (mainly motorized vehicles) is the largest source of emissions, and in San Diego, CA, it accounts for 54% of the city’s total GHG emissions [13,14].

Active travel (AT) (i.e., biking and walking to get from place to place, rather than for recreation or exercise) has been identified as a key approach to both meaningfully increase PA and mitigate GHG emissions by replacing vehicle trips with active modes [7,11,15,16,17]. In 2017, nearly 40% of all vehicle trips in the U.S. were 2 miles or less, providing a feasible opportunity to increase and maintain PA by replacing short vehicle trips [18]. AT is associated not only with increased MVPA [19,20,21,22,23,24], but also with reductions in incident CVD and cardiometabolic risk factors in large observational studies. However, the research to date has relied on self-reported AT data [25,26,27,28,29,30,31,32,33]. 

Self-reported PA data are more feasible for global surveillance, but surveys have low correlation with accelerometer-based measures and overestimate moderate PA [34,35,36]. Likewise, the proportion of U.S. adults meeting MVPA recommendations is much lower, between 5 and 45%, depending on methodology, when assessed by accelerometry, compared to 65% when measured by self-report [2,37,38,39]. Questionnaires have an advantage of capturing multiple PA domains, including PA for transportation, recreation, occupation, or housework [40]. However, combined use of accelerometer and Global Positioning System (GPS) devices to capture context-specific PA has become more common and newer methods for integrating these data have been validated for AT mode determination [41,42,43,44,45,46]. Accelerometer-measured MVPA is more strongly associated with CVD-related outcomes, compared to self-reported PA [47,48,49,50]. To our knowledge, no study has used device-based AT measures to assess relationships with CVD risk biomarkers. More accurate AT measures are critical to understanding the impact of potential interventions designed to increase AT as well as relationships between AT behaviors and health outcomes. Further, possible ethnic differences in the relationship between AT and CVD risk have not been explored, despite Hispanics/Latinos being the largest ethnic minority group in the U.S. [51,52] and having generally adverse modifiable CVD risk factor profiles compared to other racial/ethnic groups [53,54,55,56,57].

The aims of the present study were to use validated device-based measures to assess cross-sectional relationships between AT and biomarkers for CVD risk, and to compare these to associations based on self-report estimates. We hypothesized that: (i) self-reported and device-based measures of AT would vary across population subgroups, and (ii) device-measured AT would be more strongly correlated with lower body mass index (BMI), triglycerides, blood glucose and blood pressure, and higher levels of high-density lipoprotein (HDL) cholesterol, compared to self-reported measures. This study expands the literature by capitalizing on a sample with ethnic diversity from whom both objective and self-reported AT data were collected. Findings can inform investments across the public health, transportation, and climate sectors aimed at improving both individual and climate health.

## 2. Materials and Methods

### 2.1. Study Design and Participants

Community of Mine was a cross-sectional study designed to recruit an ethnically diverse sample from neighborhoods that varied by walkability and healthy food access in San Diego County, CA, to explore how disparities in environments may relate to disparities in health. Participants (N = 602) aged 35 to 80 years who had lived in their current location for at least 6 months were recruited from specific census block groups to maximize variability in built environment walkability. Recruitment was stratified by Hispanic ethnicity, sex, and age group, and the study was powered to observe relationships between built environment exposure and biomarker outcomes across ethnic groups. Participants were ambulatory without human assistance, able to read and write in Spanish or English, and willing and able to comply with study procedures. All participants provided written informed consent prior to study participation. After receiving consent, study devices (accelerometer and GPS) and questionnaires were mailed to participants. Approximately 1 week later, participants completed an in-person measurement visit where anthropometric, biomarker, and medication data were collected. They received an additional questionnaire and new study devices for a second week of wear. Devices and completed questionnaires were returned by mail after 14 total days of device wear. The University of California, San Diego’s Institutional Review Board approved the study, which was conducted in accordance with the Declaration of Helsinki (Protocol # 140510). Recruitment began in 2014 and data were collected between 2015 and 2017. Full details of the study protocol have been described elsewhere [58].

### 2.2. Exposure Variables

AT trips were assessed using data from Actigraph GT3X + accelerometer (ActiGraph, LLC; Pensacola, FL, USA) and Qstarz BT-Q1000XT GPS devices (Qstarz International Co. Ltd., Taipei, Taiwan). Participants wore both devices on a belt around their hip during waking hours for 14 consecutive days and charged the GPS device each night. Devices were screened for wear time at the clinic visit and participants were asked to re-wear devices if there were not at least 4 valid days. A valid day was defined as at least 10 h of wear time, and days that did not meet this criterion were excluded from the analysis. Accelerometer data were collected on 3 axes at 30 Hz and aggregated to minute-level files. The validated Choi algorithm excluded non-wear time as 90 consecutive zeros with a 2-min threshold during data processing [59]. To compare the sample to prior PA research, MVPA was defined as any minute that met the validated intensity threshold of 2020 counts per minute (cpm) [37]. There was no minimal bout length applied, per the current PA guidelines [2].

The GPS devices logged location, distance, speed, and time every 15 s. A validated algorithm was used to impute missing GPS data [60]. AT was identified by merging the accelerometer and GPS data using the validated Personal Activity Location Measurement System (PALMS) to classify biking, walking, and vehicle trips [42,61]. Trip criteria included a minimum distance of 100 m and 90 s duration and had to span 25 or more meters over a 1-min period. Within-trip pauses of up to 3 min were permitted to account for short stops, such as for traffic signals. PALMS designated each minute of combined data as part of a trip or not and assigned a travel mode. AT was defined as any minute spent in biking or walking trips for transportation. To exclude trips for recreational or exercise purposes, any roundtrip, defined as a start and end location within 100 m, was removed. Total minutes in non-roundtrip bike and walk trips were summed and averaged across each participant’s valid device wear days to calculate the average daily AT time. 

Self-reported PA over the previous 7 days was assessed using the long form of the International Physical Activity Questionnaire (IPAQ), a validated instrument that captures PA across the transportation, recreation, occupation, and home domains [62]. A sum of the duration and frequency of walking and cycling for transportation in the previous week was calculated and divided by 7 days to derive the daily AT exposure variable. Using recommended IPAQ scoring procedures, incomplete responses for time or days for an activity were set to missing [63]. Weekly sums for each domain were truncated at 6720 min per week, equivalent to 16 h per day. 

### 2.3. Outcome Assessments

Clinical data and fasting blood samples were collected by research clinic nursing staff at the measurement visit to generate the CVD risk outcomes: blood pressure, BMI, HDL cholesterol, triglycerides, and glucose. These biomarkers were chosen as they are metrics of metabolic syndrome, which has been strongly associated with CVD risk [64]. Systolic and diastolic blood pressures (mm/Hg) were the average of the 3 closest readings taken using a standard automated blood pressure cuff (Dinamap Accutor 7 or Dinamap V100), after a 10-min rest and before any other measures. A 4th reading was taken if 2 readings were more than 5 mmHg apart. Participants’ height and weight, without shoes, were measured twice using a stadiometer and a bariatric digital scale. BMI was calculated as average weight (kg)/height (m)^2^. Participants were asked to fast for 12 h prior to the clinic blood draw for lipid and glucose measurement. Blood was drawn into EDTA vacutainer tubes; sub-fractions were isolated by centrifugation at 4 °C and stored at −80 °C. Lipid panel components were measured at the UCSD clinical laboratory the same day as the visit. Glucose assays were performed in quality-controlled batches in plasma using the glucose oxidase method (YSI 2900 Bioanalyzer). Measured levels of the following were used for the current analysis: HDL cholesterol, triglycerides, and fasting glucose (all in mg/dL). 

### 2.4. Covariates

Participants reported demographic characteristics via self-report that included age, sex, ethnicity, race, education, income, employment status, vehicle access, and whether they had a child under the age of 18 years in the home. Binary income and education variables were created at above/below USD 55,000 and college versus no college education. Clinic staff conducted a medical history interview in which participants were asked whether they drink alcohol (beer, wine, spirits) and whether they currently smoke cigarettes or anything else. All medications were brought to the visit and written down by clinic staff. Using the IPAQ long form, total minutes per week in the recreation, work, and home domains were calculated and summed to create a variable reflecting non-transport-related PA for adjustment in statistical models. Similarly, we calculated the total weekly sitting time while at work, home, or leisure time and divided by 7 days [63]. To adjust for walkability-related self-selection to neighborhoods that may affect AT behavior, a “reasons for moving” score was created by averaging 3 survey items from an adapted scale: “desire for nearby shops and services”, “ease of walking”, and “closeness to recreational facilities” [65,66]. We adjusted for potential variability in AT that may have resulted from how long participants wore the study devices by including average daily wear time in all models [67].

To control for the effect of built environment walkability on AT, we created a dynamic walkability exposure measure using a normalized kernel density estimation (KDE) raster programmed in Python (https://www.python.org/ (accessed on 6 April 2021)). Participants’ GPS data points over their 14-day device wear period were used to generate a time-weighted activity space surface using a 200-m-bandwidth KDE function. The time-weighted activity space was then multiplied by the walkability index, comprising intersection density, residential density, and land use mix, to produce the average dynamic walkability exposure variable [68].

### 2.5. Statistical Analysis

Sample descriptive statistics present number and percentages for categorical variables and either means with standard deviation (SD) or medians and interquartile range (IQR) for skewed variables. Non-parametric Kruskal–Wallis H tests assessed differences in the median daily AT minutes across demographic subgroups [69]. We tested the presence of clustering for each outcome (without covariates) by including a random effect for the intercept at the block group level and estimated intra-class correlation (ICC). We found very small ICC values (most values were below 1%) and decided to not include random effects in our models. We used separate multivariable linear regression models to estimate associations between either device-assessed AT or self-reported AT with each outcome. To account for correlation between BP outcomes, multivariate (and multivariable) linear regression modeled systolic and diastolic blood pressure outcomes together with a single set of covariates [70]. All models adjusted for age, sex, race, Hispanic/Latino ethnicity, education, income, child <18 years in home, alcohol use, cigarette use, reasons for moving, daily sitting time, non-AT related PA, device wear time, and mean walkability exposure. The triglyceride and HDL cholesterol models were additionally adjusted for statin, antihyperlipidemic, and cholesterol-lowering medications. The blood pressure multivariate models included statins and antihypertensive medications and the glucose models adjusted for insulin, metformin, or glucose-lowering medications. Our final models did not adjust for BMI as it may mediate the relationship between AT and CVD risk factors [27,31], though we compared to models with BMI adjustment to explore this relationship. As 96% of the population had access to a vehicle, we did not include vehicle access in the models, but we did describe differences in device-assessed and self-reported AT by this variable. All models tested for effect modification by Hispanic/Latino ethnicity based on an interaction term between AT and ethnicity. For accelerometer-AT models, we conducted two sensitivity analyses including only those participants who worked or volunteered outside the home or who met the PA recommendations (defined 150 min per week of accelerometer MVPA). All analyses were conducted using Stata (Version 15.1 StataCorp LP, College Station, TX, USA).

## 3. Results

### 3.1. Sample Characteristics

Table 1 describes the 598 participants included in the analysis. There were four participants excluded due to missing outcome data. No observations exceeded the 16 h per day maximum value in the transport or recreation domains, and a total of nine observations were truncated in the work and home domains. There were eight transport walking observations that were set to missing due to incomplete responses for days per week or minutes per day.

Mean participant age was 58.8 years. The sample was 56% female, 84% had at least some college education, and fewer than half reported incomes greater than USD 55,000. On average, males were slightly older and had higher education and incomes compared to women. Participants predominantly identified as white and 42% were Hispanic overall, with a greater proportion of Hispanic/Latino women compared to men. Compliance with the device wear protocols was high in both sexes, with an average of 14 wear days and more than 14 h per day of wear time.

Participants self-reported less time in AT (median 8.6 min/day) than was assessed by accelerometry/GPS (median 14.0 min/day) and men had greater daily AT than women by both device and self-report measures. Conversely, total self-reported daily MVPA far exceeded accelerometer-measured MVPA (148.2 vs. 21.4 min/day). Sex differences were observed for MVPA as well. Accelerometer-measured MVPA was greater among men (25.7 vs. 17.8 min/day and 57% met PA recommendations vs. 45% of women), whereas women self-reported roughly 20 more minutes of MVPA per day. Fifty percent of the sample had 150 min or more of accelerometer-measured MVPA per week. However, only 31% of device-based AT met the MVPA accelerometer cut point criteria of 2020 cpm [37], so most AT time was not reflected in the total MVPA estimate. Correlation between measures of daily AT minutes was low (*ρ* = 0.115).

On average, individuals in the sample were overweight, with average fasting glucose concentration and systolic blood pressure that would meet prediabetes and stage 1 hypertension criteria, respectively. Average HDL cholesterol and triglyceride concentrations were in the normal range. Women had better risk profiles than men, with higher HDL cholesterol and lower levels of all other biomarker outcomes.

### 3.2. AT Differences across Demographic Subgroups

Table 2 presents accelerometer- and self-reported AT across demographic groups. In general, less variability and more differences across subgroups were observed with device-assessed AT. Patterns of device-based and self-reported AT were similar by age group, sex, employment status, having a child in the home, BMI, and device wear time. However, non-Hispanics had more device-based AT minutes while they self-reported less. All racial groups, with the exception of African Americans, reported less AT than measured by devices, with the largest discrepancy in Asian Americans. African American participants self-reported the highest AT minutes, however had less device-AT than White or Asian racial groups. Those with higher education and income had significantly more device-based AT, but reported less, compared to those with lower education and income. Interestingly, those without vehicle access self-reported much higher levels of AT (median 34.3 min/day) compared to those with access (median 7.1 min/day), but this difference was much smaller (1.2 min/day) and not significant when measured by devices. We did not observe significant differences in either AT measure across walkability quartiles; however, those in the least walkable quartiles had the highest device AT and the lowest self-reported AT. Device-assessed AT differed significantly by BMI category, with the obese group (34% of the sample) having the lowest levels, but a similar nonsignificant pattern was observed for self-reported AT.

### 3.3. Associations with Main Outcomes

**Figure 1:**
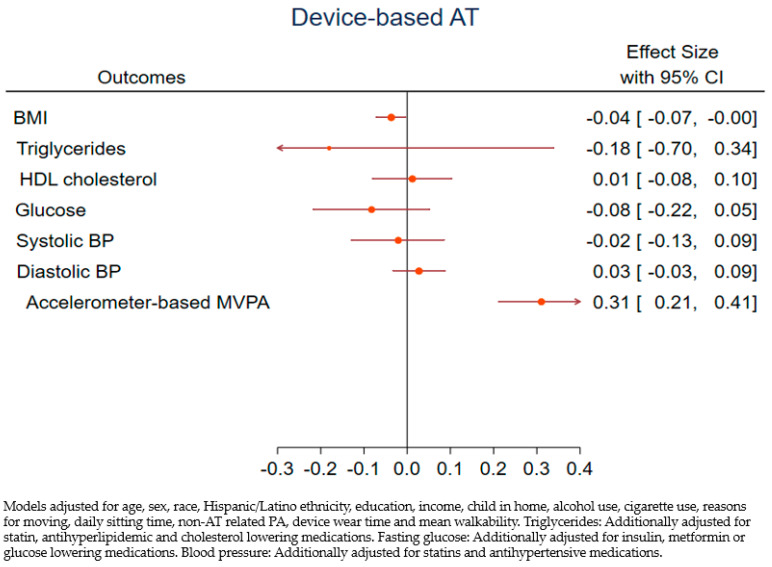
Adjusted associations between device-measured AT (min/day) and CVD risk factors.

**Figure 2:**
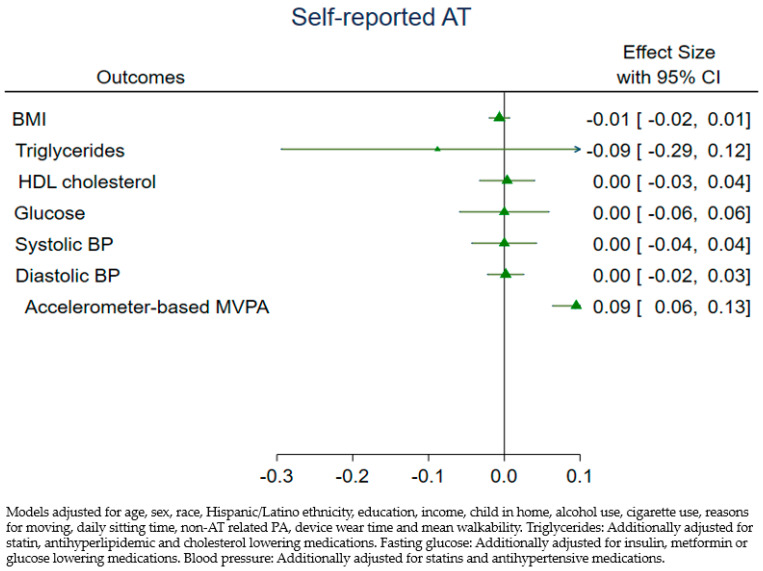
Adjusted associations between self-reported AT (min/day) and CVD risk factors.

Figure 1 and Figure 2 show the adjusted associations between device-measured and self-reported AT and CVD risk factors. Daily minutes of device-measured AT were inversely associated with BMI, triglycerides, fasting glucose, and systolic BP, though, with the exception of BMI, all confidence intervals overlapped the null value. For interpretation, a 1-min daily increase in device-measured AT was associated with a 0.04-unit decrease in BMI, for example. The relationship with HDL cholesterol was in the expected direction, while the diastolic BP estimate was contrary to expectations. We observed a similar inverse relationship between self-reported AT and triglycerides, while all other estimates were near zero (see Figure 2), and all confidence intervals included the null. Both AT measures were positively associated with total accelerometer-assessed MVPA in adjusted models; however, device-AT showed a stronger relationship (coef = 0.31, 95% CI 0.21, 0.41 versus coef = 0.09, 95% CI 0.06, 0.13). We did not find evidence of heterogeneity of effects of AT on CVD risk factors by Hispanic/Latino ethnicity with either measure.

Estimates from models that controlled for BMI to examine it as a potential mediator were similar. The direction of association for systolic BP and HDL cholesterol changed when adjusting for BMI for both AT measures; however, the estimates were very close to zero (see Table A1 and Table A2). Sensitivity analyses including only those who worked outside the home (*n* = 401, 67%) or who met the 150 min/wk MVPA recommendations as assessed by accelerometer (*n* = 300, 50%) were consistent with primary results, except that associations between device-based AT and BMI were not statistically significant.

## 4. Discussion

To date, no studies have examined associations between AT and CVD biomarkers in adults using AT derived from accelerometer and GPS devices. We identified an inverse association between device-based AT and BMI. While relationships between device-based AT measures with triglycerides, HDL cholesterol, glucose, and systolic BP were in the expected direction, effect sizes were small and all CIs included the null value. Conversely, estimates for self-reported AT were largely null, with the exception of triglycerides and MVPA, though in all cases the CIs were imprecise. Our findings were unexpected given that self-reported AT has been associated with advantageous BMI, blood pressure, cholesterol, triglycerides, and glucose or HbA1c profiles [25,30,31,71,72]. These prior studies assessed larger observational cohorts, with sample sizes ranging from ~1500 to several hundred thousand. The small sample size of the present study may explain our failure to detect associations with precision. Additionally, most prior studies utilized categorical exposure and outcome variables, making a comparison of effect sizes difficult. The present finding that device-based AT was associated as expected with BMI, while reported AT was not, provides suggestive evidence that device-based AT provides a more precise measure than self-report. This finding justifies further research on the health implications of device-based AT.

We found that device-assessed AT was higher than by self-report, which is expected given that people tend to under-report AT trips [73,74]. As hypothesized, device and self-reported AT measures differed by population subgroups, with notable differences by race, Hispanic/Latino ethnicity, education, income, vehicle access, and exposure to walkable environments. Daily MVPA between men and women differed by measurement method as women self-reported more, yet had fewer, daily MVPA minutes than men via accelerometer device. These findings call into question the validity of self-reported instruments for surveillance, assessing interventions or studying associations with health outcomes. For example, studies relying on self-report could incorrectly describe AT prevalence of racial and ethnic groups in relation to each other or fail to accurately capture changes due to AT interventions targeting specific populations.

Similar to other studies, both measures of AT were positively associated with higher total MVPA, though a stronger relationship was observed for the device-based measure [21,22,31,75,76]. Thus, AT seems to be an important contributor to overall MVPA. A recent study found that those who engaged in AT reported both more recreational MVPA and less sedentary screen time, indicating that AT may not induce compensatory reductions in other PA domains [77]. Furie and Desai found that, even among those who already met PA guidelines through leisure and work PA, higher AT was associated with lower BMI [71]. This suggests that AT has an added benefit beyond total activity.

Our sample was more active than the general population, as 50% had 150+ min/wk of MVPA using an accelerometer cut-off point of 2020 cpm [38]. Participants also had more AT than in other studies. The most recent NHANES analysis of AT trends in the U.S. reported that 14% of adults exceeded 150 min/wk of AT, whereas our study found that 31% and 33% met this criterion by accelerometer/GPS or self-report, respectively [25]. In two studies that similarly assessed AT trips for any purpose, not just for commuting, one-quarter of the sample self-reported some amount of AT, compared to 66% in the current study [27,71]. Though we aimed to maximize variability in walkability surrounding participants’ home location, recruiting from urban block groups have led to the selection of a more active sample than the general population and limited our ability to detect small associations with CVD outcomes.

It may be that active commuting is more strongly linked with health outcomes, possibly because commuting implies increased regularity. A recent systematic review of active commuting intervention studies found that increases in commute-related AT were associated with improvements in BMI, blood pressure, and total and HDL cholesterol outcomes, providing causal evidence of the link between active commuting and improved CVD risk [26]. However, it should be noted that all these studies were conducted in Europe, where AT attitudes and facilities make generalizations to the U.S. context difficult. There are several potential mechanisms by which the frequency of active commuting, regardless of overall duration, could be associated with improved CVD risk, including reductions in stress or anxiety, increased green exposure, and Vitamin D synthesis [78,79,80,81,82,83]. Daily commutes may also be easier to recall, and therefore self-reported measures of commute-specific AT may be more precise than a global AT measure, as assessed by the IPAQ [84]. Unlike our results, recent studies that measured total AT (as opposed to only trips for commute purposes) found that greater AT was inversely associated with obesity, hypertension, triglycerides, diabetes, and hypercholesterolemia [22,25,71,72]. Only Zwald et al. found a significant relationship with low HDL cholesterol, where others found no relationship, similar to this study [25,71,72]. Future device-based analyses should assess total and commute-related AT in more depth to understand whether health effects differ.

### Strengths and Limitations

To our knowledge, this is the first study to assess the relationship between AT and cardiometabolic risk factors using validated accelerometer- and GPS-derived measures of AT, in addition to self-report. The objective, continuous measures of both exposures and outcomes was a strength. Device-based AT data allowed us to further explore PA intensity during AT trips using a common accelerometer cut-off point. Combining accelerometer and GPS data enabled the detection of bicycle trips that are generally missed when only accelerometers are used. However, our AT measure could be improved by spatially matching bike and walk trips to GIS data to more accurately classify transport-only trips. Though our current method may have resulted in some exposure misclassification, we likely captured short transport trips more accurately, as evidenced by the higher daily minutes of device-measured AT compared to self-report. Compared to GPS, it has been reported that paper-based travel surveys miss 20–30% of trips [74]. Self-reported PA data are highly positively skewed and reported values may fall outside possible ranges (i.e., exceed 24 h). We followed standard IPAQ scoring protocols; however, differences in data scoring and truncation procedures may influence results across studies. The selection of a sample with high AT levels may have biased relationships between AT and the outcomes. As mentioned, the smaller sample than prior studies of AT and CVD risk factors may not have provided adequate power to detect small effects. We did not adjust for seasonal effects given that we enrolled and measured participants continuously throughout the year, and average annual temperatures in San Diego range from 58 to 70 degrees Fahrenheit [85]. Lastly, the cross-sectional design of the study limits any causal inference that may be drawn from the results.

## 5. Conclusions

Large cohort studies have found AT to be related to lower CVD incidence and mortality, indicating that this behavior warrants further investigation given the global burden of CVD [28,29,86]. The present study found a positive relationship between AT and total MVPA and a favorable association between device-assessed AT and BMI, lending support for the positive health impacts of AT. Device-based measures revealed greater daily AT time than self-report and differed with self-reported AT by population subgroups, suggesting that device-based AT could be more useful in determining which demographic groups to target with AT promotion interventions. For future study, combining accelerometers with GPS data can provide the added benefit of exploring whether AT patterns (i.e., bouts or frequency) or PA intensity during AT trips impact associations with health outcomes. Further, GPS data can elucidate the potential effects of environmental exposures during AT [41,84,87,88]. GPS data also allow a more refined classification of AT trips that could assess whether active commuting is more strongly associated with improved CVD risk than AT in general. Our results did not reveal any effect modification by ethnicity, though we did observe a discrepancy between greater self-reported AT, but less device-based AT, by Hispanic/Latino participants, compared to non-Hispanics. AT could provide a feasible behavioral target to improve CVD risk in a population that is expected to grow to nearly 30% of the U.S. population over next 40 years [51,52]. 

Importantly, results showed that AT trips can contribute to meeting MVPA recommendations, providing a needed intervention strategy given the large proportion of adults in the United States that fail to meet PA recommendations [2]. AT has the potential to address two of the most significant threats to global health by reducing both harmful emissions and individuals’ chronic disease risk [89], though doing so will require significant investment in infrastructure and policies [90]. Accurate measures are essential in future studies to understand what drives AT behaviors, to evaluate interventions designed to change them, and to assess associated health benefits. Though more expensive and burdensome, utilizing objective devices in larger studies is warranted to elucidate the true size and direction of these relationships and to confirm previously reported associations with self-reported AT.

## Figures and Tables

**Table 1:** Demographics and baseline characteristics of the study sample.

	Total *n* = 598	Female *n* = 332	Male *n* = 266
Age (years) ^a^	58.8 ± 10.9	57.5 ± 10.7	60.5 ± 10.9
College or above education	497 (83.7)	263 (79.7)	234 (88.6)
Work or volunteer outside of home	401 (67.1)	229 (69.0)	172 (64.7)
Income > $55,000	278 (48.4)	131 (40.9)	147 (57.7)
Hispanic/Latino ethnicity	248 (41.5)	151 (45.5)	97 (36.5)
Race			
White	421 (70.4)	228 (68.7)	193 (72.6)
African American	18 (3.0)	8 (2.4)	10 (3.8)
Asian	23 (3.8)	12 (3.6)	11 (4.1)
Native American	25 (4.2)	13 (3.9)	12 (4.5)
Other/Unknown	111 (18.6)	71 (21.4)	40 (15.0)
Child < 18 years in household	155 (25.9)	99 (29.8)	56 (21.1)
Access to vehicle	569 (95.8)	318 (96.4)	251 (95.1)
Current smoker	45 (7.6)	20 (6.1)	25 (9.5)
Consumes alcohol	392 (66.3)	198 (60.2)	194 (74.1)
Valid device wear days ^a^	13.8 ± 2.0	13.5 ± 2.1	14.0 ± 1.8
Average device wear time (min/day) ^a^	861.5 ± 75.0	852.5 (97.7)	867.3 (101.0)
Device measured AT (min/day) ^b^	14.0 (18.7)	13.4 (17.4)	14.7 (20.5)
Device measured MVPA during AT trips (min/day) ^b^	4.4 (11.6)	4.3 (10.5)	4.4 (12.2)
Self-reported PA by domain (min/day) ^b^			
AT	8.6 (30)	7.1 (28.6)	8.6 (39.3)
Work	0.0 (25.7)	0.0 (20.7)	0.0 (29.0)
Recreational	27.1 (61.4)	25.7 (61.4)	30.7 (62.9)
Home	38.6 (90)	50.7 (121.1)	28.6 (77.1)
Total accelerometer measured MVPA (min/day) ^b^	21.4 (28.8)	17.8 (27.3)	25.7 (27.8)
Total self-reported MVPA (min/day) ^b^	148.2 (220)	161.8 (224.3)	140.7 (186.4)
>150 min/wk of accelerometer MVPA	300 (50.2)	148 (44.6)	152 (57.1)
CVD risk factors			
BMI (kg/m^2^) ^a^	28.6 ± 5.9	28.4 ± 6.6	28.9 ± 4.8
HDL cholesterol (mg/dL) ^a^	58.7 ± 17.5	64.4 ± 34.7	51.8 ± 14.4
Triglycerides (mg/dL) ^b^	96.0 (68.5)	92.5 (65.0)	99.5 (71.0)
Systolic blood pressure (mg/dL) ^a^	131.0 ± 19.8	128.3 20.4	134.3 18.5
Diastolic blood pressure (mg/dL) ^a^	73.7 ± 10.8	70.5 ± 10.1	77.6 ± 10.2
Glucose (mg/dL) ^a^	104.5 ± 28.3	102.9 ± 29.9	106.3 ± 26.2

Values are *n*(%) unless noted, ^a^ Mean ±SD, ^b^ Median (IQR).

**Table 2:** Median minutes per day of device-measured and self-reported AT.

	Device-Assessed AT	*p*-Value	Self-Reported AT	*p*-Value
Hispanic Ethnicity		0.000		0.067
Hispanic	11.0 (15.4)		10 (40.0)	
Non-Hispanic	15.8 (21.8)		7.1 (28.6)	
Age (years)		0.589		0.745
35–49	13.1 (16.9)		5.7 (27.9)	
50–64	13.8 (17.9)		8.6 (34.3)	
65–80	15.1 (21.4)		8.6 (34.3)	
Sex		0.093		0.098
Female	13.4 (17.4)		7.1 (28.6)	
Male	14.7 (20.5)		8.6 (39.3)	
Race		0.030		0.653
White	15.2 (20.2)		8.6 (30.0)	
African American	11.4 (17.3)		13.6 (50.7)	
Asian	14.4 (28.3)		3.4 (28.6)	
Native American	9.9 (11.4)		4.3 (38.6)	
Other/Unknown	10.2 (15.6)		5.7 (30.0)	
Education		0.004		0.431
College or above	14.7 (19.4)		7.1 (30.0)	
High school or less	10.5 (15.9)		11.4 (30)	
Income		0.004		0.171
>$55,000	16.1 (22.8)		6.4 (30.0)	
≤$55,000	12.3 (16.3)		10.0 (34.3)	
Employed		0.551		0.537
Yes	13.3 (17.1)		7.1 (30.0)	
No	15.0 (20.5)		8.6 (38.6)	
Child < 18 years in household		0.458		0.443
Yes	13.4 (16.8)		7.1 (28.6)	
No	14.1 (20.2)		8.6 (34.3)	
Access to vehicle		0.666		0.000
Yes	13.9 (18.7)		7.1 (30.0)	
No	15.1 (18.4)		34.3 (111.4)	
BMI (kg/m^2^)		0.004		0.422
<25	14.7 (20.1)		10 (30.0)	
25–29.9	16.0 (20.2)		8.6 (34.3)	
≥30	11.9 (14.6)		6.4 (30.0)	
Device weartime (min/day)		0.520		0.439
≥858	13.9 (20.3)		8.6 (30.0)	
<858	14.1 (17.3)		8.6 (30)	
Walkability (KDE)		0.138		0.402
Quartile 1	15.9 (22.3)		6.1 (30.0)	
Quartile 2	11.4 (17.2)		10.0 (40.0)	
Quartile 3	14.1 (17.7)		8.6 (30.0)	
Quartile 4	13.8 (20.1)		7.1 (25.7)	

*p*-values for group differences in accelerometer and self-reported AT using Kruskal–Wallis H test.

## Data Availability

The datasets analyzed during the current study are available from the corresponding author on reasonable request.

## Appendix A

**Table A1:** Adjusted associations between device-measured AT (min/day) and CVD risk factors, with and without adjustment for BMI.

	Coef.	Std. Err.	*p*-Value	95% CI	
BMI *	−0.04	0.02	0.031	−0.07	0.00
Triglycerides, Model 1	−0.18	0.27	0.495	−0.70	0.34
Triglycerides, Model 2	−0.08	0.26	0.759	−0.60	0.44
HDL cholesterol, Model 1	0.01	0.05	0.806	−0.08	0.10
HDL cholesterol, Model 2	−0.02	0.04	0.582	−0.11	0.06
Glucose, Model 1	−0.08	0.07	0.228	−0.22	0.05
Glucose, Model 2	−0.07	0.07	0.316	−0.20	0.07
Systolic BP, Model 1	−0.02	0.05	0.700	−0.13	0.09
Systolic BP, Model 2	0.01	0.05	0.903	−0.10	0.11
Diastolic BP, Model 1	0.03	0.03	0.384	−0.03	0.09
Diastolic BP, Model 2	0.04	0.03	0.181	−0.02	0.10

* Not adjusted for BMI.; Model 1: Did not adjust for BMI in addition to all other covariates; Model 2: Adjusted for BMI in addition to all other covariates.

**Table A2:** Adjusted associations between self-reported AT (min/day) and CVD risk factors, with and without adjustment for BMI.

	Coef.	Std. Err.	*p*-Value	95% CI	
BMI *	−0.01	0.01	0.345	−0.02	0.01
Triglycerides, Model 1	−0.09	0.10	0.401	−0.29	0.12
Triglycerides, Model 2	−0.07	0.10	0.482	−0.277	0.13
HDL cholesterol, Model 1	0.00	0.02	0.840	−0.03	0.04
HDL cholesterol, Model 2	−0.00	0.02	0.920	−0.04	0.03
Glucose, Model 1	−0.00	−0.02	0.988	−0.05	0.05
Glucose, Model 2	0.00	0.03	0.949	−0.05	0.05
Systolic BP, Model 1	−0.00	0.02	0.978	−0.04	0.04
Systolic BP, Model 2	0.00	0.02	0.879	−0.04	0.04
Diastolic BP, Model 1	0.00	0.01	0.879	−0.02	0.03
Diastolic BP, Model 2	0.00	0.01	0.755	−0.02	0.03

* Not adjusted for BMI; Model 1: Did not adjust for BMI in addition to all other covariates; Model 2: Adjusted for BMI in addition to all other covariates.
